# GENDER DIFFERENCES IN COGNITIVE IMPAIRMENT OF AFRICAN AMERICAN AND HISPANIC SEXUAL AND GENDER MINORITY OLDER ADULTS

**DOI:** 10.1093/geroni/igad104.3762

**Published:** 2023-12-21

**Authors:** Austin Oswald, Karen Fredriksen-Goldsen

**Affiliations:** University of Washington, Seattle, Washington, United States; University of Washington, Seattle, Washington, United States

## Abstract

Cognitive health of sexual and gender minority (SGM) older adults with intersecting racial/ethnic minority identities is an under-researched topic. This research utilizes data from Aging with Pride: National Health Sexuality/Gender study to examine differences in health-promoting and risk factors for cognitive impairment among African American and Hispanic SGM older adults. Informed by the Health Equity Promotion Model, bivariate statistics and linear regression models were applied to better understand subgroup differences in cognitive impairment and risk and protective factors. Daily discrimination and identity stigma were positively associated with cognitive impairment for African American and Hispanic groups. Outdoor leisure was negatively associated with cognitive impairment for African Americans whereas social support was negatively associated with cognitive impairment for Hispanics. Analysis of gender differences revealed that African American men experienced significantly higher rates of identity stigma than African American women; however, the effect of identity stigma on cognitive impairment was stronger for African American women. Among Hispanics, women compared to men were significantly more vulnerable to the effects of daily discrimination and identity stigma on cognitive impairment. Regarding cognitive impairment, engaging in physical activity and outdoor leisure were significantly protective for African American men but not for women, whereas social support was significantly protective for both Hispanic men and women. These findings address a void in SGM aging research by examining racial/ethnic and gender differences in cognitive health. They highlight the need for intersectionality and subgroup differences in research to develop tailored interventions to improve cognitive health among racially/ethnically diverse SGM older adults.

